# Folkbotanical classification: morphological, ecological and utilitarian characterization of plants in the Napf region, Switzerland

**DOI:** 10.1186/1746-4269-11-13

**Published:** 2015-03-14

**Authors:** Anna Poncet, Christian R Vogl, Caroline S Weckerle

**Affiliations:** Department of Sustainable Agricultural Systems, University of Natural Resources and Life Sciences Vienna, (BOKU), Gregor-Mendel-Strasse 33, A-1180 Vienna, Austria; Institute of Systematic Botany, University of Zürich, Zollikerstrasse 107, Zürich, CH-8008 Switzerland

**Keywords:** Ethnobotany, Ethnobotanical classification, Local plant knowledge, Switzerland

## Abstract

**Background:**

Discussions surrounding ethnobiological classification have been broad and diverse. One of the recurring questions is whether classification is mainly based on the “inherent structure of biological reality” or on cultural, especially utilitarian needs. So far, studies about ethnobotanical classification have mainly been done in indigenous societies. Comparable data from industrialized countries are scarce. In this paper, folkbotanical classification data from the Napf region in central Switzerland is analysed and cross-culturally compared.

**Methods:**

Structured and semi-structured interviews were conducted with 60 adults and children chosen by random sampling. Descriptive statistics, t-tests and cultural domain analysis were used to analyze the data.

**Results:**

Close to 500 folk taxa have been documented during field work. As life-form taxa appeared tree, bush, grass, herb, flower, and mushroom. Intermediate taxa mentioned regularly were sub-categories of the life form tree and bush, i.e. conifer, deciduous tree, fruit tree, stone fruits, pomaceous fruits, and berry bush. The rank of the folk generic was by far the largest with 316 taxa (85.4% monotypical). The specific rank contained 145 taxa, the varietal 14 taxa. The 475 generic, specific and varietal folk taxa could be assigned to 298 wild growing plant species, which make up 28.13% of the local flora, and to 213 cultivated plant species, subspecies and cultivars.

Morphology, mainly life-form, fruits, leaves, and flowers, was the most important criterion for classifying plants. Other important criteria were their use (mainly edibility) and habitat (mainly meadow, forest and garden). The three criteria emerged spontaneously out of open questioning.

**Conclusion:**

The classification system of the Napf region is comparable to classification systems of indigenous societies, both in its shallow hierarchical structure and in the amount of recognized taxa.

The classification of plants was mainly guided by morphology, habitat and use. The three aspects seem to be mutually linked for certain plant groups, which results in always the same groups, independent from the different sorting criteria. Sensory perception allows for a broader explanation of the known coincidence of morphology and use groups.

## Introduction

All over the world people classify the surrounding plants and animals in folkbiological systems and appear to do this in similar ways [1]. To distinguish and thereby establish categories, to relate these categories to each other and thus establish classification systems is a deeply rooted human impulse [2]. As plants and animals are the outcome of an evolutionary process, they accordingly show regularities in morphology and behaviour. Cognitive scientists suggest, that we dispose of an innate ability to classify plants and animals, following roughly the progression of biological evolution. This classification ability helps us to perceive our environment, to memorize information about it, to reason and speculate about it and hence to interact with it e.g. [3–7].

Classifying behaviour and classification systems have been investigated by researchers of many different disciplines such as ethnology, anthropology, linguistics, cognitive sciences, zoology and botany. One of the important discussions raised in this context concerns general-purpose versus special-purpose categorization, i.e. the question whether categories are mainly based on the “inherent structure of biological reality” or on cultural, especially utilitarian needs e.g. [1, 8] pp. 2–5. Yet different types of classification, such as morphology-based and use-based systems, are often overlapping and interwoven. It has been pointed out, that a clear separation is sometimes neither possible nor appropriate [2] pp. 7–10, and that different ways of classification may also be due to intracultural knowledge variation and can thus be found within a single community [9].

However, the term “folkbiological classification system” usually designates a hierarchical classification system based on general-purpose categories only. The taxa are defined by the inherent characters of biological species which are directly distinguishable by our senses, first of all morphology, but also smell and taste of plants or the calls of animals. Although this approach neglects important aspects of categorizing plants and animals such as their use or symbolic meaning, it has the advantage that different folkbiological systems can easily be compared cross-culturally. Scientific biological classification, which is based on similar principles like folkbiological classification [3, 1, 10], is used as reference system for such comparisons. While folk classifications are valid in a restricted area only, scientific classification is consistent and globally applicable.

Most of the studies about folkbotanical classification and nomenclature have been conducted in indigenous societies. Folkbotanical studies in western countries concern either urban populations, whose knowledge about plants is poor [11–13], specialist knowledge about distinct groups of plants like trees or medicinal plants e.g. [14–16], or classification based on different habitats of plants [17]. When urban people are asked to list plants, they often produce life-form level terms like “tree”, “flower” or “grass”, whereas people from indigenous societies prefer the genus level to refer to plants [1]. The use of life-form terms indicates pronounced unfamiliarity with plants, which is also described as “devolution of knowledge” [18]. Rural populations tend to have broader plant knowledge [18–21]. However, regarding folkbotanical classification, no comprehensive study conducted in a western rural population is known to the authors.

This study aims to close this gap with an analysis of folkbotanical classification in the Napf region of Switzerland. It is part of an ethnobotanical project exploring linkages between plant diversity and local plant knowledge as a basis for applied projects in the fields of conservation, environmental awareness and education. Based on the gathered names of folktaxa of different ranks and the results of a sorting task, we outline the local folkbotanical classification system, and analyse it cross-culturally. Open questioning allowed us to exceed the general-purpose frame and to identify additional aspects important for categorizing and hence distinguishing and perceiving plants.

### Research area

**Figure 1:**
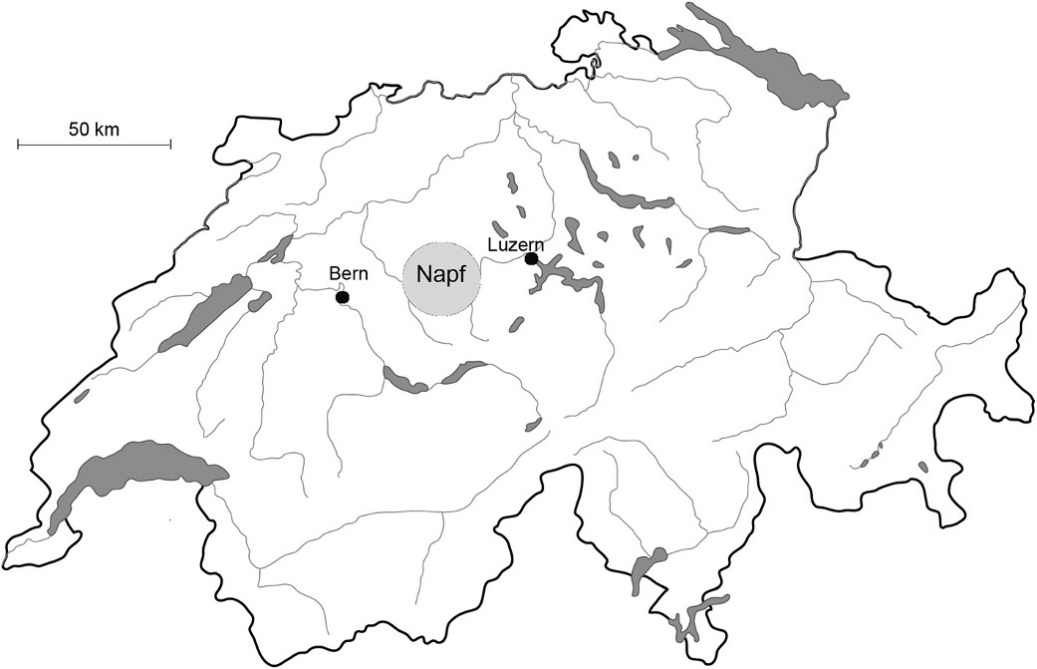
**Research area: the Napf region in Switzerland (map by first author).**

The Napf region belongs to the northern alpine foothills of Switzerland (Figure 1). It is bounded by a circular valley structure, encompasses around 500 km^2^ and touches 19 political communities. Elevation ranges between 600 and 1400 m above sea level. Annual precipitation amounts to 1736 mm and annual average temperature to 4.6°C on the centrally located summit of the Napf [22]. The underground, a molasse conglomerate, was topographically shaped by water into radially arranged valleys and ridges. The steepest and the shadowy parts are forested, whereas plainer grounds have been cleared since the 10^th^ century for agriculture. The original form of settlement is still visible: the solitary farms are surrounded by their land and a forest belt, which results in a small-scale mosaic of wood and open space. The forest is dominated by *Abies alba* Mill., *Picea abies* (L.) H. Karst. and *Fagus sylvatica* L.. Meadows and pastures are rather humid and nutrient rich, often of the type *Arrhenaterion*, *Polygono-Trisetion* or *Cynosurion*[23]. Nevertheless, because of its location between the Alps and the plains, the Napf region harbours 1063 different plant species [24, 25].

The Napf region exhibits a very rural character also in economic terms. In the nine communes lying entirely within the region, 17–73% (average 38%) of the population works in the agricultural sector, ten times more than in whole Switzerland with 3.6% [26]. Agriculture in the Napf is focused on dairy farming and upbringing of young livestock. Arable farming, which was important for subsistence until the middle of the 20^th^ century is today practiced only on the lowest and plainest grounds. A farm includes typically 10 to 20 hectares greenland plus some hectares of forest. In most of the farmer’s families at least one person has an off-farm employment. Villages are restricted to the bottom of the large valley surrounding the Napf region.

## Methods

Fieldwork was carried out by the first author during August-September 2008 and October-November 2009. A total of 60 informants living on 14 farms were interviewed. The farms were chosen by random sampling and every person living on the farm was asked for an individual interview. The interview partners comprised 33 men and boys, 8–71 years old (average 38, ±20.3), and 27 women and girls, 10–72 years old (average 36, ±19.9). All interviews were conducted in Swiss German, were recorded and are deposited at the first authors’ home. Prior to the interviews, the interview partners were informed about the project and asked for permission to record the interviews and to take pictures. After each period of fieldwork, they got a summary of the current results and the pictures taken at their homes.

The first series of interviews consisted of a freelist, followed by a semi-structured interview [27, 28]. The interviewee was asked to list all indigenous plants he or she could think of (“*Säg mer aui iiheimische Pflanze, wo der i Sinn chöme*”) and was then asked for possible uses of the listed plants.

In order to get higher order categories, a pile sorting task was subsequently performed with the first 36 of the 60 freelist informants [29]. Since it is important, that the interviewees know all the plants which they have to sort, the 43 most frequently listed plants were used and their names printed on small cards. We did not use plant pictures, as they may produce groupings based on superficial morphological similarity [16]. With the question “Which plants belong together?” (“*Weli Pflanze ghöre zäme?*”) the informants were asked individually to sort the 43 cards in as many piles as they wanted to. Each card could be used only once. They were then encouraged to explain their sorting. The 36 pile sortings were done by 20 men and 16 women, aged from 11 to 72 years (average 45, ±19.1).

Species identification was done by means of transect walks and participatory observation [30]. Voucher specimens were taken in presence of the informants, identified according to the Flora Helvetica [31] and deposited at the herbarium of the Natural Museum of Lucerne (NMLU). Cultivated plant species were identified at the spot and not vouchered. If they do not figure in the Flora Helvetica, the nomenclature follows the publications of the Swiss edition-lmz [32–34].

The folkbiological terminology used to analyse the data follows Berlin [1]. According to his general principles, folkbiological systems are organized in a hierarchical structure of 6 taxonomic ranks: kingdom, life-form, intermediate, generic, specific, varietal. He defines the ranks as follows: The highest rank of the kingdom contains the two categories plant and animal, although they are not always explicitly named. Life-form taxa form rather large groupings of perceptually similar folk genera, usually based on a small number of biological characters. The intermediate rank is situated between generic and life-form rank. Intermediate taxa assemble up to a dozen of similar folk generics. The generic rank appears to be the largest and most important rank. Folk generics are “the smallest fundamental biological discontinuities easily recognized in any particular habitat” [1] p. 53. They can be recognized without close study and often bear a short, single name. About 80% of them are monotypic (no subclasses). Polytypic generic taxa are split in two or more specific taxa. The recognition of a specific taxon requires closer examination. It differs in a few characters from other specific taxa of the same generic taxon and bears typically a complex, descriptive name. Folk varietal taxa occur seldom and mainly among plant species that are intensively manipulated, e.g. crop plants of farming societies. Regarding the terminology, it is important not to confuse folk taxa with scientific taxa. A folk generic, for example, corresponds usually with both, the scientific rank of genus and species, sometimes also with taxa of other scientific ranks. Scientific and folk systems are coherent only within themselves. Therefore, throughout this article the term „plants“ is used instead of „species“ if the concerned folk taxa corresponds with more than one systematic rank.

For outlining a comparable folkbotanical classification system we assigned the gathered plant names from freelists and pile sortings to the most appropriate rank of Berlin’s folk classification by applying his rank characteristics. Important for the definition of generic and specific taxa were first of all the names of the plants, but also the criteria of “easy recognition” versus “need for closer examination” (see above), as well as the order of the plant names in the freelists. If different names were used for a plant, the most frequently mentioned and most concise name was used for the analysis. For example, the two cabbage varieties *Brassica oleracea* L. convar*. capitata* var*. sabauda* L. and *B. oleracea* L. var*. capitata* L. were occasionally both called *“Chool”*. However, as most people use *“Chool”* only for the former and call the latter *“Chabis”*, the two varieties appear in our list as two different folk generics. Intermediate terms were gained from pile sorting, whereby general-purpose groupings based on morphological traits were considered only. Important classification criteria other than morphology are presented in the second part of the results.

The data was organized with Access 2007, t-tests were performed with SPSS 16.0 and Cultural Domain Analysis (Cultural Consensus and Hierarchical Clusteri.ng) was done with Anthropac [35, 27].

## Results

### Numbers and levels of folk taxa and folk taxonomic overview

The 60 freelists contained 7 to 108 items (mean 44.6, ±26.5). In total 458 different folk taxa were listed, including 14 fungi. The most frequently listed species was *Taraxacum officinale* aggr*.*, followed by *Rumex obtusifolius* L. and *Rubus fruticosus* aggr. (Table 1).

**Table 1:** **The 43 most frequently listed plants, subsequently used for the pile sorting**

German name	Scientific name	Family	Times mentioned (n = 60)
Löwenzahn	*Taraxacum officinale* aggr.	Asteraceae	52
Blacke	*Rumex obtusifolius* L.	Polygonaceae	43
Brombeere	*Rubus fruticosus* aggr.	Rosaceae	36
Weisstanne	*Abies alba* Mill.	Pinaceae	34
Rottanne	*Picea abies* (L.) H. Karst.	Pinaceae	33
Himbeere	*Rubus idaeus* L.	Rosaceae	33
Apfelbaum Distel	*Malus domestica* Borkh.	Rosaceae	33
Schwarzer Holunder	*Sambucus nigra* L.	Adoxaceae	33
Brennessel	*Urtica dioica* L.	Urticaceae	33
Ahorn	*Acer pseudoplatanus* L.	Sapindaceae	32
Rotklee	*Trifolium pratense* L. s.l.	Fabaceae	32
Weissklee	*Trifolium repens* L.	Fabaceae	32
Spitzwegerich	*Plantago lanceolata* L.	Plantaginaceae	31
Buche Linde	*Fagus sylvatica* L.	Fagaceae	29
Hahnenfuss	*Ranunculus* spp.	Ranunculaceae	29
Birnbaum Margrite	*Pyrus communis* L.	Rosaceae	29
Linde	*Tilia platyphyllos* Scop., *T. cordata* Mill.	Malvaceae	28
Margrite	*Leucanthemum vulgare* Lam.	Asteraceae	28
Hasel	*Corylus avellana* L.	Betulaceae	26
Schlüsselblume	*Primula elatior L., P.veris* L.	Primulaceae	26
Heubeere	*Vaccinium myrtillus* L.	Ericaceae	25
Gänseblümchen	*Bellis perennis* L.	Asteraceae	25
Vogelbeere	*Sorbus aucuparia* L.	Rosaceae	23
Eiche	*Quercus robur* L.	Fagaceae	23
Zwetschgenbaum	*Prunus domestica* L.	Rosaceae	23
Sonnenblume	*Helianthus annuus* L.	Asteraceae	22
Distel	*Cirsium* spp., *Sonchus* spp.	Asteraceae	21
Farn	Polypodiidae	e.g. Aspleniaceae, Woodsiaceae	21
Wilde Erdbeere	*Fragaria vesca* L.	Rosaceae	21
Sauerampfer	*Rumex acetosa* L.	Polygonaceae	20
Breitwegerich	*Plantago major* L. s.l.	Plantaginaceae	20
Kirschbaum	*Prunus avium* L.	Rosaceae	20
Stechpalme	*Ilex aquifolium* L.	Aquifoliaceae	20
Esche	*Fraxinus excelsior* L.	Oleaceae	19
Meertrübeli	*Ribes rubrum* L.	Grossulariaceae	18
Hagebutte	*Rosa* spp. (e.g. *R. canina* L., *R. pendulina* L. )	Rosaceae	18
Birke	*Betula pendula* Roth	Betulaceae	18
Frauenmänteli	*Alchemilla* spp.	Rosaceae	16
Efeu	*Hedera helix* L.	Araliaceae	16
Knaulgras	*Dactylis glomerata* L.	Poaceae	16
Englisch Raygras	*Lolium perenne* L.	Poaceae	16
Salbei	*Salvia officinalis* L.	Lamiaceae	16
Pfefferminze	*Mentha x piperita* L.	Lamiaceae	15

The semi-structured interviews, the transect walks, the participatory observation and the pile sorting task yielded roughly 40 additional names of taxa, also morphologically defined higher order taxa, which were important for the outlining of the folk taxonomy. Of the 475 generic, specific and varietal folk taxa (listed in Table 2) 222 are wild growing, 191 are cultivated on fields or in homegardens and 62 are wild growing but also cultivated. The wild growing folk taxa could be assigned to 298 plant species, which make up 28.13% of the local flora with 1063 species. The cultivated folk taxa were assigned to 213 cultivated plants (species, subspecies and cultivars).

**Table 2:** **Generic, specific and varietal taxa**

Generic taxa		Herbarium	Specific taxa		Herbarium	Varietal taxa		Herbarium
Scientific_name	Local_name	Number*	Scientific_name	Local_name	Number*	Scientific_name	Local_name	Number*
*Abies* spp./*Picea* spp.	Tanne, Tanneböim		*Abies alba* MILL.	Tanne, Wiisstanne				
			Abies nordmanniana	Nordmannstanne,	080930 4–3			
			(STEVEN) SPACH	Normannstanne	080930 4-4			
			*Picea abies* (L.) H. Karst.	Rottanne, Fichte				
			*Picea pungens* ENGELM.	Blautanne	080930 4–1			
*Acer* spp.	Ahorn, Ahorne		*Acer platanoides* L.	Spitzahorn	080930 4–2			
			*Acer pseudoplatanus* L.	Bärgahorn, Ahorn	090730 4–3 090730 4–4			
*Achillea millefolium* agg.	Schafgarbe, Schofgarbe	090708 2–1 090708 2–2 090730 9–7 090730 9–8 100801 1–1 100801 1–2						
*Aegopodium podagraria* L.	Boumtropfe, Baumtropfe							
*Aesculus/Castanea*	Kaschtanie, Kaschtanieboum, Cheschtene, Cheschtenebuum, Cheschtele, Cheschteleboum, Chegele, Chegeleboum		*Aesculus hippocastanum* L.	Rosscheschtene, Rosskaschtanie, Söicheschtene				
			*Castanea sativa* MILL.	Edukaschtanie				
*Agaricus campestris* L. ex FR.	Fäudschampinjoo, Schampinjoo, Schampinjoopüuz,							
*Ajuga reptans* L.	Kriechender Günsel							
*Alchemilla alpina* agg.*/A. conjuncta* agg.	Silbermänteli, Süubermänteli, Siubermänteli							
*Alchemilla* spp. (e.g. *A.vulgaris* L. agg./A. *glabra* agg./A. *hybrida* agg.)	Frouemänteli, Frauemänteli, Frouemantu, Frauenmantel, Toumänteli	090815 1–3 090815 1–4 100801 1–8 100801 1–9						
Algae (eukaryot.)	Auge							
*Allium cepa* L.	Zibele, Zwibele, Zwiebel							
*Allium porrum* L.	Louch, Lauch							
*Allium sativum* L.	Chnoblouch, Chnobli, Knoblauch							
*Allium schoenoprasum* L.	Schnittlouch		*Allium schoenoprasum* L.	Wiude Schnittlech				
			*Allium schoenoprasum* L. (cult.)	Schnittlouch, Schnittlauch, Schnittlech				
*Allium* sp. (cult. ornamental)	Allium							
*Allium ursinum* L.	Bärlouch, Bärlauch, Bärlouchchrut, Rämsere							
*Alnus* spp.	Erle		*Alnus incana* (L.) MOENCH	Erle, Roterle, normali Erle	081004 2–3 081004 2–4 081004 3–3 081004 3–4			
			*Alnus viridis* (CHAIX) DC.	Erle, Schwarzerle (!)				
*Althaea officinalis* L.	Eibisch							
*Althaea rosea* (L.) CAV.	Malve, Stockrose							
*Alyssum maritimum* (L.)	Alüssum							
LAM.								
*Amanita muscaria* (L. ex FR.) LAMARCK	Flüügepiuz, Flöigepiuz, Fliegepüuz							
*Amanita phalloides* (VAILL. ex FR.) LINK	Chnouebletterpiuz							
*Anemone hupehensis* LEMOINE								
*Anemone nemorosa* L.	Geisseblüemli, Geisseglöggli, Buschwindröschen, Wucherblüemli							
*Antennaria dioica* (L.) GAERTN.	Musenöörli							
*Anthyllis vulneraria* L. s.l.								
*Antirrhinum majus* L.	Löiemüli							
*Apiaceae (Anthriscus/ Chaerophyllum/Heracleum/ Angelica)*	Chirbele, Kerbel, Chirbelenarte		*Angelica sylvestris* L.	Chirbele, Chirbele für Pfiiffe	080930 3–8			
			*Anthriscus sylvestris* (L.) HOFFM.	Chirbele, Wisechirbele, Wisecherbu, Chirbelestängu				
			*Chaerophyllum villarsii* W. D. J. KOCH	e Chirbele, Boumtropfe	091019 4–1 091019 4–2 091019 4–3			
			*Heracleum mantegazzianum* SOMMIER et LEVIER	Chärbu				
			*Heracleum sphondylium* L. s.l.	Bäretaupe, Bäretatze, Bäretööpe, wiudi Chirbele, Schärlech, Schärlig	080816 1–1 090730 6–8 090815 1–5 091003 1–1 100801 3–5 100801 3–6			
			*Myrrhis odorata* (L.) SCOP.	Chörblichrut				
			*Anthriscus sylvestris* (L.) HOFFM.	Chirbele, Wisechirbele, Wisecherbu, Chirbelestängu				
			*Chaerophyllum villarsii* W. D. J. KOCH	e Chirbele, Boumtropfe	091019 4–1 091019 4–2 091019 4–3			
			*Heracleum mantegazzianum*	Chärbu				
			SOMMIER et LEVIER					
			*Heracleum sphondylium* L. s.l.	Bäretaupe, Bäretatze, Bäretööpe, wiudi Chirbele, Schärlech, Schärlig	080816 1–1 090730 6–8 090815 1–5 091003 1–1 100801 3–5 100801 3–6			
			*Myrrhis odorata* (L.) SCOP.	Chörblichrut				
*Apium graveolens* L. var. *rapaceum* (MILL.) GAUD.	Sellerii, Sällerii, Säuerii							
*Aquilegia vulgaris* L.	Akelei, Aupe-Akelei							
Armoracia rusticana	Meerrättich, Meerrätech							
*Arnica montana* L.	Arnika							
*Artemisia absinthium* L.	Wermuet, Mueterchrut							
*Arum maculatum* L.	Aaronstab							
*Aruncus dioicus* (WALTER)	Bockbart, Geissbart							
FERNALD								
*Aster* sp. (cult.)	Aschter, Aschterli							
*Atropa belladonna* L.	Touchirschi							
*Avena sativa* L. s.l.	Haber, Hafer							
*Begonia* sp.	Begonie							
*Bellis perennis* L.	Gänseblüemli, Gänseblümchen, Gänsegismeli, Geisseblüemli, Waseblüemli, Wasebürschteli, Margritli	090730 6–3 090730 6–4 090815 2–1 090815 2–2 100801 3–3 100801 3–4						
*Berberis julianae* C. K. SCHNEID.	Chrüzdorn, Chrüzlitorn	091019 2–2 091019 2–3						
*Beta vulgaris* L. var. crassa (ALEF.) WITTM.	Fueterrüebe, Rüebe, Ruebe							
*Beta vulgaris* L. ssp. *vulgaris* var. *conditiva* ALEF.	Rande							
*Beta vulgaris* L. ssp. *vulgaris* var. *flavescens* DC.	Chrutstile, Chrutschtiu							
*Betula pendula* ROTH	Birke, Birche	090730 3–1 090730 3–2						
*Boletus* spp.	Steipiuz, Steipüuz							
*Borago officinalis* L.	Borretsch							
*Brassica napus* L. var. o*leifera* (MOENCH) DELILE	Raps							
*Brassica oleracea* L. convar. *acephala* var. *gongylodes* L.	Choleräbli, Chouräbli							
*Brassica oleracea L.* convar*. botrytis* (L.) *ALEF.* var. *botrytis* L.	Bluemechou, Bluemechöli, Bluemchöli							
*Brassica oleracea* L. convar. *botrytis* (L.) ALEF. var. *italica* PLENCK	Broggoli							
*Brassica oleracea* L. convar. *capitata* var. *sabauda* L.	Chou, Chööli, Kohl, Wirz							
*Brassica oleracea*var. *gemmifera* DC.	Rööselichou, Rosechou, Rööselichööli							
*Brassica oleracea* L. var. *capitata* L.	Chabis, Chool		*Brassica oleracea* L.convar. *capitata* var. *alba* L.	Chabis, Wiisschool				
			*Brassica oleracea* L.convar. *capitata* var. *rubra* L.	Rotchool, Rotchabis, Blauchabis, Blauchrut				
*Brassica rapa* L. var. *esculenta* L.	Runggle, Herbschtrüebe							
*Bryophyta*	Miesch, Moos							
*Buxus sempervirens*L.	Buchs, Buchsi	100328 1–3 100328 1–4						
*Cactaceae*	Kaktus							
*Calendula officinalis* L.	Ringublueme							
*Calluna vulgaris* (L.) HULL	Brüüsch, Erika, Erikabüsch	090815 3–1 090815 3–2 090815 3–3 100801 8–1 100801 8–2						
*Caltha palustris* L.	Sumpfdotterblueme, Dotterblueme, Guggerblueme, Bachbumele	090510 2–4 090510 2–5 090708 1–1 090708 1–2						
*Calystegia sepium* (L.) R. BR./ *Convulvulus arvensis* L.	Winge		*Calystegia sepium* (L.) R. BR.	Winge Wicke	090702 2–3 090702 2–4 100801 7–1 100801 7–2			
			*Convulvulus arvensis* L.	Acherwinde				
*Campanula cochleariifolia* LAM.*/ C. rhomboidalis* L.	Gloggeblüemli, Gloggeblueme, Wüudi Gloggeblueme	090815 3–8 090815 3–9 090818 1–1 090818 1–2 090821 1–1 090821 1–2						
*Cannabis sativa* L.	Hanf							
*Cantharellus cibarius* FR.	Eierschwumm, Eierschwümm, Eierschwämm							
*Capsella bursa-pastoris* (L.) MEDIK.	Hirtetäschli, Hirtechrut	100801 3–2						
*Cardamine pratensis* L.	Wiesenschaumkraut, Bettseierli, Knöterich	090510 2–6 090510 2–7 090510 2–8						
*Carex* spp.*/Juncus* spp.*/ Molinia* spp.	Ried, Riedgras, Sauergräser, Lische, en Art Schilf	081005 5–5 081005 5–6	*Carex* sp.	Lische, Segge, Seggenart, kantiges Gras	081007 2–1 081007 2–2			
			*Juncus effusus* L.*/J. inflexus* L.	Binse, Riedröhrli, Schnittlouchgras	090815 8–3 090815 8–4 090821 2–1 090821 2–2 090821 2–3 090821 2–4			
			*Molinia caerulea* (L.) MOENCH/*M. arundinacea* SCHRANK	Ried, Riedströi	081007 3–1 081007 3–2			
*Carex* sp. (cult.)	Ziergras							
*Carlina/Cirsium/Sonchus*	Dischtle, Dischtli, Tischtle	081004 4–1 081004 4–2 081004 4–3 081005 4–1 081005 4–2 090828 1–1	*Carlina acaulis* ssp. *caulescens* (LAM.) SCHÜBL. et G. MARTENS	Siuberdischtle, Süuberdischtle, Silberdischtle, chlini Dischtle, Edudischtle				
			*Cirsium acaule* SCOP.	nideri Dischtle, chlini roti Dischtle	081005 3–1 081005 3–2			
			*Cirsium arvense* (L.) SCOP.	Dischtle wo sech über Wurzle verbreite, Dischtle mit Uslöifer, Chratzdischtle, Ackerkratzdistel	081001 1–1 081001 1–2 081004 1–1 081004 1–2 081004 1–3 100801 9–1 100801 9–2			
			*Cirsium oleraceum* (L.) SCOP.	Bachdischtle, Muniseckeli, Sumpfchrut	080930 1–1 080930 1–2 090730 2–1 090730 2–2 091019 1–1 091019 1–2			
			*Cirsium palustre* (L.) SCOP.	höchi Dischtle, Stängudischtle, grossi Dischtle	081005 4–1 081005 4–2			
			*Cirsium vulgare* (SAVI) TEN.	sehr stachligi höchi Dischtle, importierti Dischtle	081004 1–4 081004 1–5 081005 4–1			
			*Cirsium vulgare* (SAVI) TEN.*/C. palustre* (L.) SCOP.	normali Dischtle, angeri Dischtle (>< Milchdischtle), höchi Dischtle, Dischtle wo Rosette mache u höch u violett blüije	081007 5–1 081007 5–2 081007 5–3 081007 5–4			
			*Onopordum acanthium* L.	Esusdischtle				
			*Silybum marianum* (L.) GAERTN.	Mariedischtle				
			*Sonchus asper* HILL/*S. arvensis* L.s.l.	Milchdischtle, Müuchdischtle, Wasserdischtle	080930 5–1 080930 5–2 081001 2–1 081001 2–2 081007 4–1 081007 4–2 090730 3–3 090730 3–4 090730 3–5 100801 4–1 100801 4–2			
*Carpinus betulus* L.	Wiissbueche Hagebueche							
*Carum carvi* L.	Chümi							
*Centaurea montana* L.	Chräijeschnäbu, grossi blaui Blueme							
*Centaurium erythraea* RAFN	Tuusigguudichrut							
*Chaenomeles japonica* (THUNB.) LINDL. ex SPACH.	Füürbusch							
*Chelidonium majus* L.	Warzechrut							
*Chenopodium album* L.	Mäubele	100801 4–10 100801 10–1						
*Chenopodium bonus-henricus* L.	Guter Heinrich							
*Cichorium intybus* L.	Wägwarte							
*Cichorium* spp., *Lactuca sativa* L., *Valerianella locusta* (L.) LATERR.	Salat, Salot		*Cichorium endivia* L. var. *latifolium* HEGI	Endivie, Ändivie, Ändivi				
			*Cichorium intybus* L. partim	Zuckerhuet				
			*Cichorium intybus* L. partim (red)	Schiggoree				
			*Lactuca sativa* L. var. *capitata* L.	Chopfsalat, Chopfsalot				
			*Lactuca sativa* L. var. *capitata* L.	Isbärg				
			*Lactuca sativa* subsp. *crispa* (L.) SCHÜBL. & G.MARTENS	Schnittsalat				
			*Valerianella locusta* (L.) LATERR. (cult.)	Nüssler, Nüssli, Nüsslisalot				
*Clematis* sp. (cult.)	Klematis							
*Colchicum autumnale* L.	Herbschtzitloose							
*Consolida ajacis* (L.) SCHUR	Rittersporn							
*Convallaria majalis* L.	Meieriisli							
*Coprinus comatus* (O.F. MÜLL. ex FR.) PERS.	Tintling							
*Cornus mas* L.	Tierliboum, Kornelkirsche							
*Cornus sanguinea* L.	Hartriegel							
*Cornus* sp. (cult. orn.)	Kornus							
*Corylus avellana* L.	plant: Hasle, Haslere, Haslerete, Hasel, Haselstude, Hasustude, Hasunussstude, Hasustruuch; fruit: Hasunuss		*Corylus avellana* L.	plant: Hasle, Haslere, Haslerete, Hasel, Haselstude, Hasustude, Hasunussstude, Hasustruuch; fruit: Hasunuss	090730 4–5 090730 4–6 100328 1–5 100328 1–6 100328 1–7 100801 2–1 100801 2–2			
			*Corylus avellana* L. (cult. ornamental)	Zierhasu				
*Crataegus monogyna* JACQ.	Wiissdorn, Eggedorn	100801 1–10 100801 1–11						
*Craterellus cornucopioides* (L. ex FR.) PERS.	Totetrumpete							
*Crepis biennis* L.	Pippou, Pippau, Pippaum, Wisepippou, Wiesenpippau, Sibegringe, gäubi steichigi Blüemli	090702 5–7 090702 5–8 090730 8–1 090730 8–2 090813 1–1						
*Crocus* sp.	Krokus, Krokussli							
*Cucumis sativus* L.	Gurke							
*Cucurbita maxima* DUCH.	Chürbis							
*Cucurbita pepo* L.	Zuggetti							
*Cyclamen* sp.	Ziklame							
*Cydonia oblonga* MILL.	Chüttene, Quitte, Quitteboum							
*Cypripedium calceolus* L.	Froueschue							
*Dahlia x hortensis*	Daalie							
*Daphne mezereum* L.	Säidelbascht							
*Daucus carota* L.	Wüudi Mööre	090730 9–9 090730 9–10						
*Daucus carota* L. ssp. *sativus* (Hoffm.) ARCANG.	Rüebli							
*Dianthus barbatus* L.	Stiinägeli, Steinägeli, wüudi Steinägeli, Nägeli, Neuke							
*Dicentra spectabilis* (L.) LEMAIRE	Frouehärzli							
*Digitalis* spp.	Fingerhuet							
*Digitaria/Echinochloa*	Hirse	100801 4–11 100801 4–12						
*Echinacea* sp.	Sunnehuet, Sonnenhut							
*Epilobium angustifolium* L.	Widerööseli	100801 6–3 100801 6–4						
*Equisetum arvense* L./E. *sylvaticum* L.	Bettseiergras, Chatzeschwanz, Chatzeschwänz, Chatzefarn, Chatzeschtile, Fuchsschwanz, Schachtuhaum, Isechrut, Rainfarn	080910 5–1 080910 5–2 080910 5–3 081004 3–1 081004 3–2 081005 5–3 081005 5–4 090730 2–5 090730 2–6 090815 8–5 090815 8–6 090815 8–7 090815 8–8 100801 1–14 100801 1–15 100801 7–5 100801 7–6						
*Eriophorum* spp.	Wougras, Bärgmanndli	090708 4–1 090708 4–2						
*Erodium cicutarium* (L.) L'HER.	Reierschnabu							
*Eruca vesicaria* ssp. s*ativa* (MILL.) THELL.	Rucola							
*Euonymus europaea* L.	Pfaffehüetli							
*Euphorbia* sp.	Woufsmiuch							
*Euphrasia* spp. e.g. *E. rostkoviana* HAYNE	Ougetroscht	090815 3–4 090815 3–5						
*Fagus sylvatica* L.	Bueche, Buche, Rotbueche	090730 4–7 090730 4–8						
*Filipendula ulmaria* (L.) MAXIM.	Mädesüess, Geisleiterebluescht, Wiesenbocksbart,	080930 3–3 080930 3–4 090815 8–1 090815 8–2 100801 1–3 100801 1–4						
*Foeniculum vulgare* MILL. var. *azoricum* (MILLER) THELLUNG	Fänchu, Fenchu							
*Fomes* sp.	Piuz a tote Böim							
*Forsythia* x *intermedia* ZABEL	Forsiizie							
*Fragaria* spp.	Äbberi, Ärbeeri, Ärdbeeri, Ebbeeri, Erdbeere		*Fragaria ananassa* (WESTON) LOIS et al.	Gartenäbbeeri				
			*Fragaria vesca* L.	wiudi, wüudi, wildi Äbberi, wildi Ebberi, chliini Äbbeereli, Waudäbbeeri, roti Beeri am Port, Äbbeeribletter, Äbbeerichrut	090815 4–4 090815 4–5			
*Fraxinus excelsior* L.	Esche, Ösche	090730 4–1 090730 4–2						
*Fuchsia* sp.	Fuchsie							
*Galanthus nivalis* L.	Schneglöggli, Schneglogge							
*Galeopsis tetrahit* L.	Gluure, Luege, Houzaan	080904 1–6 090702 3–3 090702 3–4 090730 1–11 090730 1–12 100801 2–7 100801 2–8						
*Galinsoga ciliata* (RAF.) S.F. BLAKE	Franzosechrut							
*Galium aparine* L.	plant: Chliibere, Labchrut, Chläbere; fruits: Chläblüüs	090702 1–1 090702 1–2 100801 5–3 100801 5–4						
*Galium mollugo* L. agg.	wisses Chrütli	081005 7–1 081005 7–2 081005 7–3						
*Galium odoratum* (L.) SCOP.	Waudmeischter							
*Gentiana* spp.	Enzian, Änzian, Änziane		*Gentiana asclepiadea* L.	Stänguenzian				
			*Gentiana clusii* E. P. PERRIER et SONGEON/ *G. acaulis* L.	Grosse Änzian				
			*Gentiana lutea* L.	Gäubi wo si d Würze stäche				
			*Gentiana verna* L.	Chline Änzian, chli Enzian				
*Geranium robertianum* L. s.str./G. *pyrenaicum* Burm.f.	Storcheschnabu, Hasechrut	080904 1–2 090708 2–5 090708 2–6 090730 7–1 090730 7–2 100801 1–12 100801 1–13 100801 5–1 100801 5–2						
*Geum rivale* L.	Buebehösli, Bachnelkenwurz, Bachtschötteli, Kaputschinerli							
*Gladiolus communis* L.	Gladiole							
*Glechoma hederacea* L. s.l.	Gundelrebe							
*Hedera helix* L.	Eföi, wi Ahornbletter wo so ufewachse	090815 5–1 090815 5–2						
*Helianthus annuus* L.	Sunneblueme							
*Helianthus tuberosus* L.	Topinambur, Furzchnoue							
*Helleborus niger* L.	Chrischtrose							
*Hepatica nobilis* SCHREB.	Läberblüemli							
*Hippophae rhamnoides* L.	Sanddorn, Sangdorn							
*Hordeum vulgare* L. s.l.	Gärschte, Gerste			*Hordeum vulgare* L. s.l.	Summergärschte (2 rows of seeds)			
				*Hordeum vulgare* L. s.l.	Wintergärschte (4–6 rows of seeds)			
*Humulus lupulus* L.	Hopfe							
*Hyacinthus orientalis* L.	Hiazinte							
*Hydrangea macrophylla* (THUNB.) SER.	Hortensie							
*Hypericum perforatum* L./*H. maculatum* CRANTZ s.l.	Johannis-Chrut	081005 2–1 081005 2–2 090730 9–3 090730 9–4 100801 7–7						
*Hyssopus officinalis* L.	Isop							
*Ilex aquifolium* L.	Paume, Paumestöck, Stächpaume, Stächpalme, Muttipaume (branches with prickle-less leaves)	091019 2–1 100328 1–2						
*Imleria badia* (FR.) VIZZINI	Maroneröörling							
*Impatiens* spp.	Springchrut		*Impatiens glandulifera* ROYLE	Chinesisches Springchrut, Himalaya Chlepfchrut, Roserots Springchrut	080904 1–1 100801 5–5 100801 5–6			
			*Impatiens noli-tangere* L./*I. parviflora* DC.	Rüerminidaa, Rühr-mich-nicht-an, Gäubs Springchrut	090730 7–3 090730 7–4			
*Iris* sp. (cult. ornamental)	Iris, Schwärtlilie							
*Juglans regia* L.	Nussboum, Nussbuum, Grosse Nussboum, Boumnuss, Baumnuss							
*Juniperus communis* L. s.l.	Wachouder, Wachouderstruuch, Wacholder, Räckhoudere	080930 4–5 080930 4-6						
*Juniperus sabina* L.	Sefi	100328 1–9 100328 1–10						
*Knautia arvensis* (L.) COULT./ *K. dipsacifolia* KREUTZER	Witweblueme, wüudi Skabiose, Blaui ir Ökowise	091003 2–1						
*Laburnum anagyroides* MEDIK.	Guudräge, Goudräge							
*Lamium galeobdolon* (L.) L. s.l./*L. maculatum* (L.) L./*L. purpureum* L./*Prunella vulgaris* L.	Toubnessle, Ummuchrut		*Lamium maculatum* (L.) L./*L. purpureum* L./ *Prunella vulgaris* L.	Beiichrüttli, Ummuchrut	090702 4–1 090702 4–2 090702 4–5 090702 4–6			
*Larix decidua* MILL.	Lärche, Lerche							
*Lavandula* sp.	Lavändu, Lavendel							
*Lemna* sp.	Wasserlinse							
*Leontopodium alpinum* CASS.	Eduwiiss							
*Lepidium sativum* L.	Chressi							
*Leucanthemum vulgare* agg.	Margrite, Margritli, Zantihansblueme	090730 6–1 090730 6–2 090730 6–7						
*Leucojum vernum* L.	Märzeglöggli, Meiglöggli							
*Levisticum officinale* W.D.J. KOCH	Maggichrut							
*Ligustrum vulgare* L.	Liguster							
*Lilium martagon* L.	Türkebund, Türkebndlilie							
*Lilium* sp. (cult. ornamental)	Amaryllis, Lilie							
*Linum usitatissimum* L.	Liinblüemli, Flachs							
*Lobelia erinus* L.	Lobelie							
*Lonicera xylosteum* L.	Bäsehouz, Vogubeeri, die mit de rote Beeri							
*Lupinus polyphyllus* LINDL.	Lupine							
*Lycopersicon esculentum* MILL.	Tomate							
*Lycopodium annotinum* agg./*L. clavatum* L.	Bärlapp							
*Lysimachia punctata* L.	Felberich							
*Macrolepiota procera* (SCOP. ex FR.) SINGER	Parasol							
*Malus* spp.	Öpfuboum, Öpfubuum, Öpfubaum, Öpfu, Öpfle, Öpfel		*Malus domestica* BORKH.	Öpfuboum, Öpfubuum, Öpfubaum, Öpfu, Öpfle, Öpfel				
			*Malus sylvestris* (L.) MILL.	wildi Öpfel				
*Malva neglecta* WALLR.	Chäslichrut	090702 4–9 090702 4–10						
*Malva sylvestris* L.	Mauve, Malve, Chäslichrut							
*Matricaria* spp.	Kamiue, Kamüue, Kamille		*Matricaria chamomilla* L.	Kamiue, Kamüue, Kamille	100801 4–3 100801 4–4			
			*Matricaria discoidea* DC.	Kamiuedings, Strahlenlose Kamille	081001 3–1 081001 3–2 081001 3–3			
*Medicago sativa* L.	Luzärne, Lüsärne							
*Melissa/Monarda*	Melisse		*Melissa officinalis* L.	Zitronemelisse, Melisse, wiudi Melisse				
			*Monarda didyma* L.	Goudmelisse, Guudmelisse				
*Mentha* spp.	Münze		*Mentha aquatica* L.	wiudi Münze				
			*Mentha longifolia* (L.) HUDS.	wiudi Münze, wüudi Münze, Rossmünze, Chatzemünze	081001 4–1 081001 4–2 090707 1–3 090707 1–4 100801 1–6 100801 1–7			
			*Mentha spicata* L. (cult.)	Krauseminze				
			*Mentha spicata* L. "Marokko"	Libanesischi Pfäffermünze, Libanesische Pfäffermünz				
			*Mentha suaveolens* EHRH.	Öpfumünze	091019 3–1 091019 3–2			
			*Mentha suaveolens* EHRH. "Variegata"	Ananasmünze				
			*Mentha x piperita* L.	Pfäffermünz, Pfefferminze, normali Münze				
			*Mentha x piperita* L. var. c*itrata* (EHRH.) BRIQ.	Orangschemünze				
*Mespilus germanica* L.	Mischple							
*Montia perfoliata* (DONN ex WILLD.) HOWELL	Portulak							
*Morchella* sp.	Morchle							
*Muscari* sp.	blaui Primeli							
*Myosotis* spp.	Vergissmeinnicht							
*Narcissus poeticus* L.	Narzisse							
*Narcissus pseudonarcissus* L.	Oschterglogge, Oschterglöggli, Apriuglogge, Narzisse							
*Nasturtium officinale* R. BR.	Brunnekresse							
*Nigritella nigra* (L.) RCHB. agg.	Männertröi							
*Nymphaea/Nuphar*	Seerose		*Nuphar lutea* (L.) SM.	Gäubi Seerose				
			*Nymphaea* sp.	Seerose				
*Ocimum basilicum* L.	Basilikum							
*Oenothera biennis* agg.	Nachtcherze							
*Onobrychis viciifolia* SCOP.	Esparsette							
*Ononis spinosa* L. s.l./ *O. repens* L.	Hauhechel							
*Orchidaceae*	Orchideä		*Dactylorhiza maculata* agg.	wüudi Orchidee				
			*Orchidaceae* (cult.)	Orchidee, Orchideä				
*Origanum majorana* L.	Mejoran							
*Origanum vulgare* L.	Doste, Dost, Meiran, en Art Münze im Trochene	090707 1–5 090707 1–6 090730 9–1 090730 9–2						
*Origanum vulgare* L. (cult.)	Oregano							
*Paeonia* spp.	Pfingschtrose							
*Papaver sominferum* L./*P. dubium* L. s.str./ *P. rhoeas* L.	Moon	100801 4–9						
*Paris quadrifolia* L.	Einbeere							
*Passiflora* sp.	Passionsblueme							
*Pelargonium* spp.	Granium, Grani, Geranie, Granie, Graninie							
*Petasites albus* (L.) GAERTN.	Peschtwurz, Rebarbere am Wäg							
*Petroselinum crispum* (Mill.) NYM.	Peterli, Petersilie							
*Phallus impudicus* L. ex PERS.	Stinkmorchle							
*Phaseolus* spp.	Boone		*Phaseolus coccineus* L.	Füürboone				
			*Phaseolus vulgaris* L. ssp. *vulgaris* var. *nanus* (L.) ASCHERS	Buschboone				
			*Phaseolus vulgaris* L. ssp. *vulgaris* var. *vulgaris*	Stangeboone				
*Phragmites australis* (CAV.) STEUD.	Schiuf							
*Physalis alkekengi* L.	Latärneblueme, Latärme							
*Pimpinella saxifraga* L.	Bibernelle							
*Pinus cembra* L.	Arve							
*Pinus strobus* L.	Weimuet							
*Pinus sylvestris* L.	Fööre, Dääle, Dääl							
*Pisum sativum* L.	(Ärbs)		*Pisum sativum* L.	Ärbsli, Ärbs				
*Pisum sativum* L. ssp. a*rvense* (L.) ASCH. & GRAEBN.	Ärbs							
*Pisum sativum* L. convar. *axiphium* ALEF.	Chifu, Chefe							
*Plantago* spp.	Wägerich, Wägerech		*Plantago lanceolata* L.	Spitzwägerich, Spitzwägerech, Spitzwegerich, Wägerich mit länge Bletter	080904 2–2 090815 1–1 090815 1–2			
			*Plantago major* L. s.l.	Breitwägerich, Breitwägerech, Wägerich mit churze Bletter	080904 1–3 090815 2–3 090815 2–4			
			*Plantago media* L.	Mittlerer Wegerich				
*Poaceae*	Gras, Grasarte, Greser, Schmäle, Schmale, Schmaale		*Agrostis stolonifera* L.	Struussgras				
			*Alopecurus myosuroides* HUDS.	Acherfuchsschwanz				
			*Alopecurus pratensis* L.	Fuchsschwanz, Wisefuchsschwanz				
			*Anthoxanthum odoratum* L.	Ruchgras, Gruchgras, Geruchgras				
			*Arrhenatherum elatius* (L.) J. & C. PRESL	Fromentau, Fromental, französisches Reigras	080910 2–1 080910 2–2			
			*Briza media* L.	Zittergras	090708 4–3 090708 4–4			
			*Bromus erectus* HUDS. s. str.	Trespe, Mareilihoor	081007 7–1 081007 7–2			
			*Bromus hordeaceus* L.	weichi Treschpe, weichi Träschpe				
			*Cynosurus cristatus* L.	Kammgras				
			*Dactylis glomerata* L.	Chnougras, Chnoulgras, Knaulgras, Chnaulgras, Chnöiugras, Chnüttuschmale				
			*Elymus repens* (L.) GOULD	Riischgras, Quecke, Spitzgras, Schnüergras	081007 6–1 081007 6–2			
			*Festuca* spp.	(Schwingu Schwingel)		*Festuca arundinacea* SCHREB. s.l.	Rohrschwingu	
						*Festuca pratensis* HUDS. s.l.	Wiseschwingu, Wiseschwingel, Wiesenschwingel, Schwingu	
						*Festuca rubra* L. agg.	Rotschwingu	
			*Holcus lanatus* L.	wolliges Honiggras, wolligs Honiggras, Wollgras	080910 4–3 080910 4–4			
			*Lolium perenne* L./*L. multiflorum* LAM/ *L. multiflorum* LAM. var. w*esterwoldicum* WITTM.	Reigras		*Lolium multiflorum* LAM.	italiänisches Reigras, italiänisch Reigras	080910 4–1 080910 4–2
.						*Lolium multiflorum* LAM. var. *westerwoldicum*WITTM	Westerwoldisches Reigras	
						*Lolium perenne* L.	Änglisch Reigras, änglisches Reigras, änglisches Reegras, Reigras, Rischgras, Weidlgras, französisches Reigras	090730 8–3 090730 8–4
			*Nardus stricta* L.	Burschtgras				
			*Phleum pratense* agg.	Timotee				
			*Poa* spp.	Rischpegras, Spitzgras, Fänschgras		*Poa annua* L.	Eijärigs Rischpegras, Fänschgras, Spitzgras	081007 1–1 081007 1–2
						*Poa pratensis* agg.	Wiserischpegras, meerjärigs Rischpegras, Spitzgras	
						*Poa trivialis* L. s.l.	Gemeins Rischpegras, Gemeini Wiserischpe, Gmeini Rischpe	
			*Trisetum flavescens* (L.) P. BEAUV.	Goudhaber, Guudhaber, wüude Haber	090815 3–10 090815 3–11			
*Polygonatum multiflorum* (L.) ALL.	Salomonssigu	090730 1–13 090730 1–14						
*Polygonum bistorta* L.	Zaanbürschtli, Zangbürschtli, Wisechnöterich	090730 2–3 090730 2–4						
*Polygonum persicaria* L.	pfirsichblättriger Knöterich, Chnöterich, Knöterich	080930 4–7 080930 4–8						
*Polypodiaceae (Dryopteris* spp*./ Athyrium/ Oreopteris/ Pteridium)*	Farn, Farne	080930 2–1	*Dryopteris filix-mas* (L.) SCHOTT/*Athyrium filix-femina* (L.) ROTH/ *Oreopteris limbosperma* (ALL.) HOLUB	Wurmfarn, Stockfarn, Fäderfarn	081004 2–1 081004 2–2 081005 5–1 081005 5–2 090815 4–1 090815 4–2 090815 4–3 090815 6–1 090815 6–2 090821 2–5 090821 2–6			
			*Pteridium aquilinum* (L.) KUHN	Adlerfarn Stängufarn angere Farn (>< Stockfarn)	081005 5–7 081005 5–8 090815 7–1 090815 7–2			
			undef.	chliises Farn im Wald				
*Populus tremula* L.	Zitterpapple, Papple, Eschpe, Espe							
*Potentilla anserina* L.	Gänsefingerkraut							
*Potentilla erecta* (L.) RAEUSCH.	Bluetwurz							
*Primula elatior* (L.) L./ *Primula veris* L. s.str.	Schlüssublueme, Schlüssublüemli, Schlüsseli		*Primula elatior* (L.) L.	Schlüssublüemli	090510 2–1 090510 2–2 090510 2–3			
*Primula veris* L. s.str.	Ehrezäicheli							
*Prunus armeniaca* L.	Aprikose							
*Prunus avium* L.	Chriesi, Chriesboum, Chriesiboum, Chriesibaum, Chirschi, Chirschiboum, Chirschibuum, Chirschboum, Kirsche		*Prunus avium* L.	wiudi Chirschi, wildi Chriesi	090730 1–7 090730 1–8			
			*Prunus avium* L. (cult.)	Chriesi, Chriesboum, Chriesiboum, Chriesibaum, Chirschi, Chirschiboum, Chirschibuum, Chirschboum, Kirsche		*Prunus avium* L. (cult. black varieties)	Schwarzi Chirschiböim	
						*Prunus avium* L. (cult. black variety)	Rigi-Chriesi	
						*Prunus avium* L. (cult. red varieties)	Roti Chirschiböim	
*Prunus domestica* L.	Zwätschgeboum, Zwätschgebuum, Zwätschgebaum, Zwätschge							
*Prunus domestica* L. ssp. *prisca* BERTSCH	Ziberliboum, Ziberli, Ziiberli	081007 8–1 081007 8–2						
*Prunus domestica* L. ssp. *syriaca* (BORKH.) JANCH.	Mirabelle							
*Prunus insititia* L.	Pflüümliboum, Pfluumeboum, Pflüümli, Pfluume, Zwätschge							
*Prunus persica* (L.) BATSCH	Pfirsich							
*Prunus spinosa* L.	Schwarzdorn							
*Pseudotsuga menziesii* (MIRB.) FRANCO	Duglasie, Duglase, Duglas							
*Psilocybe semilanceata* (Fr.) P. KUMM.	Psilos, magic mushrooms							
*Pyrus communis* L.	Bireboum, Birebuum, Birebaum, Bire, Birne							
*Quercus robur* L.	Eiche	090730 1–9 090730 1–10						
*Ranunculus* spp.	Hanefuess, Hänifuess		*Ranunculus aconitifolius* L.	wiisse Hanefuess				
			*Ranunculus* spp. (flowering yellow)	Hanefuess, gäubi Ankeblüemli, Ankeblueme, chliini gäubi Blüemli	090730 6–5 090730 6–6 100801 1–5	*Ranunculus acris* L. s.l.	angere Hanefuess (>< kriechend), scharfe Hanefuess	080904 2–1
						*Ranunculus repens* L.	kriechender Hanefuess, breite Hanefuess	
*Raphanus/ Sinapis*	(Sänf)		*Raphanus raphanistrum* L.	Wiisse Sänf	100801 4–5 100801 4–6 100801 4–7 100801 4-8			
			*Sinapis arvensis* L.	Gäube Sänf	100801 7–3 100801 7–4			
*Raphanus sativus* L. var. *niger* (MILL.) J. KERN	Rättich, Rettech							
*Raphanus sativus* L. var. s*ativus* (red varieties)	Radiisli							
*Rheum rhabarbarum* L.	Rebarbere							
*Rhinanthus alectorolophus* (SCOP.) POLLICH	Klappertopf, Chlappertopf, Klappergras	090708 3–1 090708 3–2						
*Rhododendron ferrugineum* L./*R. hirsutum* L.	Auperose							
*Rhododendron* sp. (cult.)	Rhododendron							
*Ribes rubrum* L./*R. nigrum* L.	Trübeli, Meertrübeli		*Ribes nigrum* L.	Schwarzi Trübeli, Cassi, Cassis				
*Ribes rubrum* L.	Roti Trübeli, Trübeli, Meertrübeli, Johannisbeeri							
*Ribes uva-crispa* L.	Chrusle, Stachubeeri, Stachelbeere							
*Ribes x nidigrolaria*	Joschta							
			*Robinia pseudoacacia* L.	Robinie, fautschi Akazie				
*Rosa* spp.	Rose		*Rosa* spp. (cult.)	Rose, Roseböimli				
			*Rosa* spp. (wild, e. g. *R. canina* L.*, R. pendulina* L*.*)	wiudi Rose, Hagrose, Hagrööseli, Hagbuttestruuch, Hagebutte (fruits)				
*Rosmarinus officinalis* L.	Rosmarin, Rosmarii							
*Rubus fruticosus* agg.	Brombeeri, Brommerli, Brumeli, Brummbeeri		*Rubus fruticosus* agg.	fruits: wüudi Brombeeri, Brumeli; plant: Dörn, Brammertörn, Brambeeritörn, Brombeeritörn, Brumelidörn, Dornbüsch	090730 1–1 090730 1–2			
*Rubus fruticosus* agg. (cult.)	Brombeeri, Brommerli, Brummbeeri							
*Rubus idaeus* L.	Himbeeri, Himbeere, Himbeeristude, Himpeli, Himpi, Hinti	090730 1–5 090730 1–6 100801 2–3 100801 2–4						
*Rudbeckia hirta* L.	Rudbeckia							
*Rumex acetosa* L.	Surampfer, Surampfere, Sauerampfer, Suurchrut, Guggersuur	090702 5–1 090702 5–2						
*Rumex obtusifolius* L.	Blacke, Blacki, Wiseblacke, Dittiblacke, Grossblättriger Ampfer	100801 3–1 100801 5–7						
*Salix* spp.	Wide, Weide	090730 4–11 090730 4–12						
*Salvia* spp.	Saubei, Salbei, Saubine, Salbine		*Salvia nemorosa* L.	Prachtsaubei				
			*Salvia officinalis* L.	Saubei, Salbei, Saubine, Salbine				
			*Salvia officinalis* L. "Tricolor"	Ziersaubei				
			*Salvia pratensis* L.	Wisesaubei				
			*Salvia splendens* SELLOW ex ROEM. & SCHULT.	Salvia				
*Sambucus* spp.	Holunder, Houder, Houdere, Houler, Houerstock, Houderestruuch		*Sambucus nigra* L.	schwarze Holunder, schwarze Houder, blaue Houder, schwarze Houer, schwarzi Houdere, Houler	090730 5–3 090730 5–4			
			*Sambucus racemosa* L.	rote Houder, rote Houer, rote Houler, roti Houdere	090730 5–1 090730 5–2 100801 2–5 100801 2–6			
*Sanguisorba minor* SCOP. s.str.	Wisechnopf							
*Sanicula europaea* L.	Scharniggu							
*Satureja hortensis* L.	Boonechrut, Bohnenkraut							
*Scilla* sp.	Meiglöggli							
*Secale cereale* L.	Rogge							
*Sempervivum tectorum* L.	Oktoberli, Huswürze							
*Senecio jacobaea* L.	Chrüzchrut, Jakobskreuzkraut							
*Silene dioica* (L.) CLAIRV./*S. flos-cuculi* (L.) CLAIRV.	Nägeli		*Silene dioica* (L.) CLAIRV.	Fleischblueme, Nägeli				
			*Silene flos-cuculi* (L.) CLAIRV.	Chropfnägeli, Harznägeli, Kuckuckslichtnelke, Seifechrut				
*Silene vulgaris* (MOENCH) GARCKE s.l.	Liimchrut							
*Solanum tuberosum* L.	Härdöpfu, Härdöpfel, Kartoffel							
*Solidago canadensis* L./*S. gigantea* AITON	Goldruete, Goudruete Goldrute							
*Sorbus aria* (L.) CRANTZ	Mählbeeri							
*Sorbus aucuparia* L.	Vogubeeri, Vogubeeriboum, Vogelbeeri, Vogelbeere, Eberesche, Gürmsch, Gürmschli	090730 4–9 090730 4–10						
*Sparassis crispa* (WULFEN) FR.	Krause Glucke							
*Spinacia oleracea* L.	Spinat, Spinet							
*Staphylea pinnata* L.	Bibernüssli	091019 1–3 091019 1–4						
*Stellaria media* (L.) VILL.	Hüenerdarm, Hüenderdarm, Vogumiere, Vögelichrut	080930 5–3 080930 5–4						
*Symphoricarpos albus* (L.) S.F. BLAKE	Struuch mit wiisse Bböueli							
*Symphytum officinale* L.	Wallwurz, Wauwürze, Beinwell							
*Syringa vulgaris* L.	Flider							
*Tagetes* sp.	Tagetes							
*Tanacetum vulgare* L.	Reinfarn	090730 9–5 090730 9–6 100801 6–1 100801 6–1						
*Taraxacum officinale* s.l. agg.	Söiblueme, Süiblueme, Löwezaan, Chrottepösche							
*Taxus baccata* L.	Eibe	100328 1–8						
*Thuja occidentalis* L.	Thujahaag							
*Thymus* spp.	Timian, Thümian	081005 6–1 081005 6–2 090815 3–6 090815 3–7	*Thymus serpyllum* L. agg.	wiude Tümian, wüude Timian				
*Thymus vulgaris* L.	Tümian, Gartentümian							
*Thymus vulgaris* L. "fragrantissimus"	Orangschetimian							
*Tilia platyphyllos* SCOP./ *T. cordata* MILL.	Linde, Lindeboum, Lindebaum, Linge; flowers: Lindeblüete, Lindebluescht	090702 3–1 090702 3–2	*Tilia cordata* MILL.	Winterlinde				
			*Tilia platyphyllos* SCOP.	Summerlinde				
*Tradescantia* sp.	Gottesauge							
*Tragopogon pratensis* L. s.l.	Bockbart							
*Trifolium/Lotus/Medicago/Oxalis*	Chlee		*Lotus corniculatus* agg.	Gäubchlee, Gälchlee, gäube Chlee, Schotechlee, Steichlee, Steechlee, Steinklee, Hornchlee	090702 2–1 090702 2–2 090708 2–3 090708 2–4			
			*Medicago lupulina* L./*Trifolium campestre* SCHREB.	Hopfechlee, Gäubchlee	090707 1–7 090707 1–8			
			*Oxalis acetosella* L.	Suurchlee, Waudchlee, Hasechlee	090510 3–1 090510 3–2 090510 3–3 090708 4–5 090708 4–6			
			*Trifolium alexandrinum* L.	Alexandrinerchlee				
			*Trifolium hybridum* L. s.str.	Baschtardchlee				
			*Trifolium incarnatum* L.	Inkarnatchlee				
			*Trifolium pratense* L. s.l./ *T. medium* L./ *T. repens* L.	Chleeblettli, Chleeblüemli, vierbletterigs Chleeblatt				
			*Trifolium pratense* L. s.str. / *T. medium* L.	Rotchlee, Chleeblüemli, Mattechlee	090702 5–5 090702 5–6			
			*Trifolium repens* L.	Wiss-Chlee, Weissklee, Chleeblüemli	080904 1–4 090702 5–3 090702 5–4			
*Triticum aestivum* L.	Weize, Winterweize, Wäize							
*Triticum spelta* L.	Dinku, Dinkel, Chorn							
*Trollius europaeus* L.	Ankebäueli, Ankebäui, Töni, Moosbouele							
*Tropaeolum majus* L.	Stigüferli, Kapuzinerli							
*Tulipa* sp. (cult.)	Tuupe, Tulpe							
*Tussilago farfara* L.	Zitröseli, Zitteröseli, Windröseli, Huflattich	090510 1–1 090510 1–2						
*Ulmus glabra* HUDS.	Uume, Ulme							
*Urtica dioica* L*.*	Brönessle, Bränessle, Nessle							
*Vaccinium myrtillus* L./ *V. corymbosum* L.	Höibeeri, Heidubeeri, Heidelbeeri, Heidelbeere		*Vaccinium corymbosum* L.	Heidubeeri				
			*Vaccinium myrtillus* L.	Höibeeri, Höibeeristude, Höibeeri im Waud, wiudi Höibeeri	090818 2–1 090818 2–2 090818 2–3 100801 6–5 100801 6–6			
*Vaccinium vitis-idae* L.	Fuchsbeeri	090815 9–1 090815 9–2 090815 9–3						
*Valeriana officinalis* agg.	Baudrian							
*Verbascum* spp.	Chünigs-Cherze, Chönigs-Cherze, Wuublüemli, Wuublueme, Wolleblüemli							
*Veronica chamaedrys* L./*V. filiformis* SM./ *V. persica* POIR	Chatzenöögli, Chatzenöigli, Chatzeöögli, Chatzeöigli, Chatzenouge, Ehrenpreis					090702 4–3 090702 4–4 090702 4–7 090702 4–8 100801 11–1 100801 11–2 100801 11–3		
*Veronica officinalis* L.	Eerepriis							
*Viburnum* spp.	Schneebau		*Viburnum lantana* L.	Wüude Schnebau, Vogubeeri, wolliger Schnebau				
			*Viburnum opulus* L.	angere Schnebau (>< wolliger), Schneebau	090730 1–3 090730 1–4			
*Vicia cracca* L. s.l./*V. sepium* L.	en Art Ärbsgwächs, Wicke	090707 1–1 090707 1–2						
*Vinca major* L.	Stritte							
*Viola* spp.	Veieli	090510 4–1 090510 4–2	*Viola odorata* L.	Veieli				
			*Viola reichenbachiana* BOREAU/ *V. riviniana* RCHB.	Tubechropf, Hundsveieli				
*Viola tricolor* agg.	Stifmüeterli, Stöifmüeterli							
*Viscum album* L. s.l.	Mischtle, Mischteler, Mistel							
*Vitis vinifera* L.	Trube, Räbe							
*x Triticosecale*	Tritical, Triticau							
*Zea mays* L.	Meis, Mäis							

*all the herbarium numbers correspond to the herbarium NMLU, collection A. Poncet.

Taxonomy (for names and rank assignment see Tables 2 and 3): The plant kingdom was given by the freelist question. The informants listed all kinds of plants including ferns and mosses, in six cases also fungi. As life-form taxa appeared tree (*Boum*), bush (*Stude, Struuch*), grass (*Gras, Greser*), herb (*Chrüttli, Chrütter*), flower (*Blüemli*), mushroom (*Piuz, Pilz)*. The intermediate taxa were difficult to grasp, because groupings of generic taxa which are not life-forms are often motivated by other than morphological criteria (see below). However, intermediate general purpose taxa which appeared regularly during the pile-sorting task were sub-categories of the life form tree (conifer, deciduous tree, fruit tree, stone fruits, pomaceous fruits) and bush (berry bush). The rank of the folk generic is by far the largest: it contains 316 taxa. Of these taxa 85.4% are monotypical. In total 186 generic taxa correspond to genera in scientific classification (many of them also monotypical in the region), 110 taxa correspond to lower scientific ranks such as species or even subspecies (e.g. blueberry, *Vaccinium myrtillus L.* or beetroot, a cultivar of *Beta vulgaris* L*.* ssp*. vulgaris*) and 20 taxa correspond to higher scientific ranks up to classes (e.g. fern, *Polypodiidae*). The specific rank contains 145 taxa, the varietal 14 taxa. Of the specific taxa 96 correspond to the scientific rank of the species, 30 to lower, 19 to higher ranks. In 11 cases, the difference between two specific taxa refers to their cultivation status, e.g. garden strawberry and wild strawberry.

**Table 3:** **General purpose higher order taxa**

Life-form taxa		Intermediate taxa (1st order)		Intermediate taxa (2nd order)	
Local name	English term	Local name	English term	Local name	English term
Boum	tree				
		Naduboum, Nadelboum, Tanne	Conifer		
		Loubboum	Deciduous tree		
		Obschtboum	Fruit-tree		
				Steiobscht	Stone fruits
				Chärnobscht	Pomaceous fruits
Stude, Struuch	Bush, shrub				
		Beeristude	Berry bush		
Gras, Greser	Grass				
Chrüttli, Chrütter	Herb				
Blüemli, Blueme	Flower				
Piuz, Pilz	Mushroom				

Children and adolescents up to 20 years old (n = 20) listed significantly less plants (mean 24.95, ±18.72) than interviewees above 20 (n = 40, mean 54.35, ±24.29; t-test p <0.001). Their lists also contained proportionally more generic taxa (mean 85.79%, ±9.41) than the lists of the adults (mean 72.8%, ±12; t-test p <0.001). No difference in the listed number of plants could be detected between younger adults (21–40 years old, n = 13), middle-aged adults (41–60 years old, n = 18) and older adults (above 60 years, n = 9).

### Criteria used by the interviewees for pile sorting

The interview partners split the cards into 4 to 16 (mean 9.2, ±2.67), in total 405 groups. The used criteria concerned almost exclusively morphology, use and habitat. One group could get more than one attribute, like, for example, the frequently formed group of the berries which was defined by both morphology and use. The two criteria of this group appear in the statement of an informant, which explained, that they are “small, round and edible things”. Morphology influenced the forming of groups in 228 cases, use in 218 cases and habitat in 107 cases. In 41 cases other criteria were used like the seasons (e.g. spring flowers), the importance for insects, esthetical reasons (nice or not nice to look at, beauty), similarities of plant names or no explanation.

During the pile sorting most informants used two or more criteria. Only four of the 36 interview partners sorted the plants consistently by one single criterion: twice by habitat, once by use and once by morphology.

**Figure 2:**
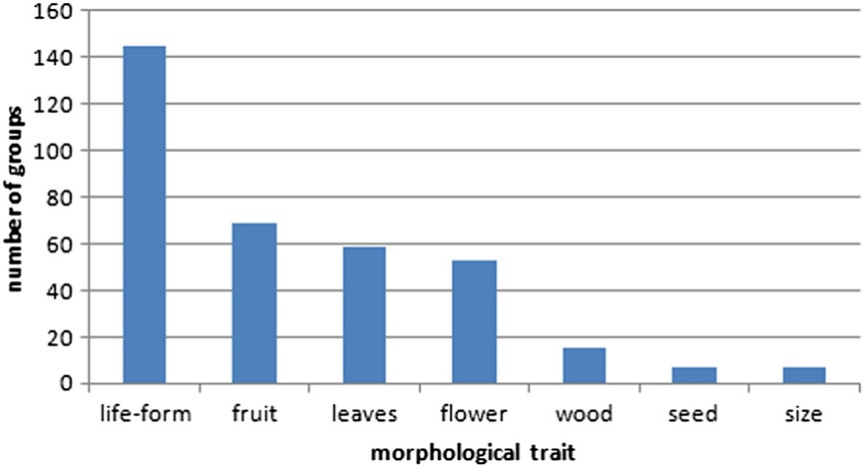
**Frequency of different morphological traits used for forming plant groups (n = 36).**

Morphological criteria were: the size and form of the plant, if the plant climbs or not, if the plant is woody or not, properties of the wood, form and properties of the leaves (e.g. prickly, evergreen), form, size and colour of the flowers, form and size of the fruits and seeds. The most often mentioned trait was the life-form, followed by fruit, leave and flower (Figure 2).

**Figure 3:**
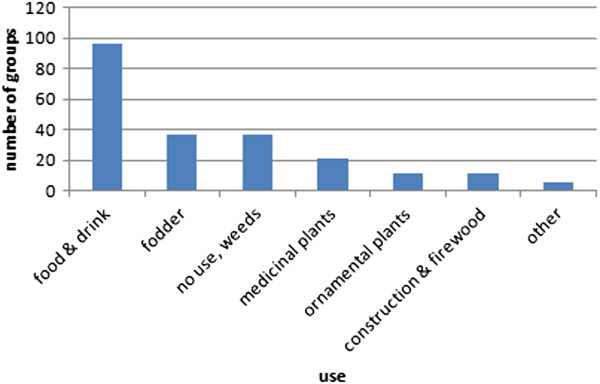
**Frequency of different types of uses relevant for forming plant groups (n = 36).**

The most important use relevant for the sorting of plants was edibility (Figure 3). Berries were put together because they are used for desserts, jam and syrup, fruit trees because of their similar use for eating, juice and schnapps, wild or garden herbs because of their use in salads, as spices or for herbal tea. Other groups contained fodder plants for the cattle, medicinal or ornamental plants. Tree groups were sometimes explained with the argument that their wood can be used for construction or as firewood. In 37 cases the formed group was declared as “plants of no use” or “useless weeds”. They were also noted as use-groups in the sense that they are defined by non-use.

**Figure 4:**
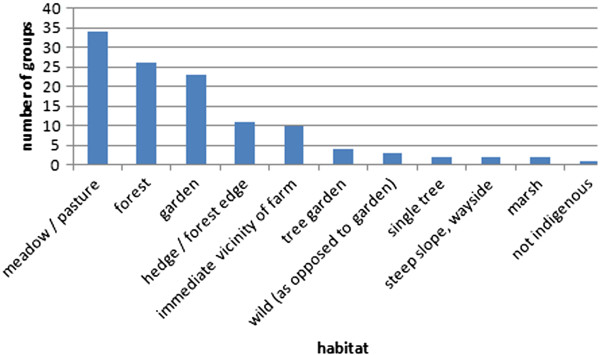
**Frequency of habitats relevant for forming plant groups (n = 36).**

During the pile sorting task 11 habitats were used to explain the groups (Figure 4). The most often mentioned habitats “meadow/pasture”, “forest” and “garden” were further divided into sub-habitats. As sub-habitats for meadows and pastures appeared: extensive (nutrient poor) meadow, intensive (nutrient rich) meadow, artificial meadow, forest meadow, meadow with sour soil, wet pasture. Sub-habitats of the forest were: Lothar-forest (patches of forest where most of the trees were knocked over in 1999 by the storm “Lothar”) and the differentiation into trees and brushwood. Sub-habitats of the garden were: “Pflanzblätz” (a vegetable or fibre crop plot in some distance to the farm) and the differentiation into plants at the fence, in the path and as boundary.

Habitats additionally mentioned during the semi-structured interviews but not used for the sorting task were: field, alpine pasture, brookside, pond and “places where many people walk”.

### Cultural domain analysis of the pile sorting

**Figure 5:**
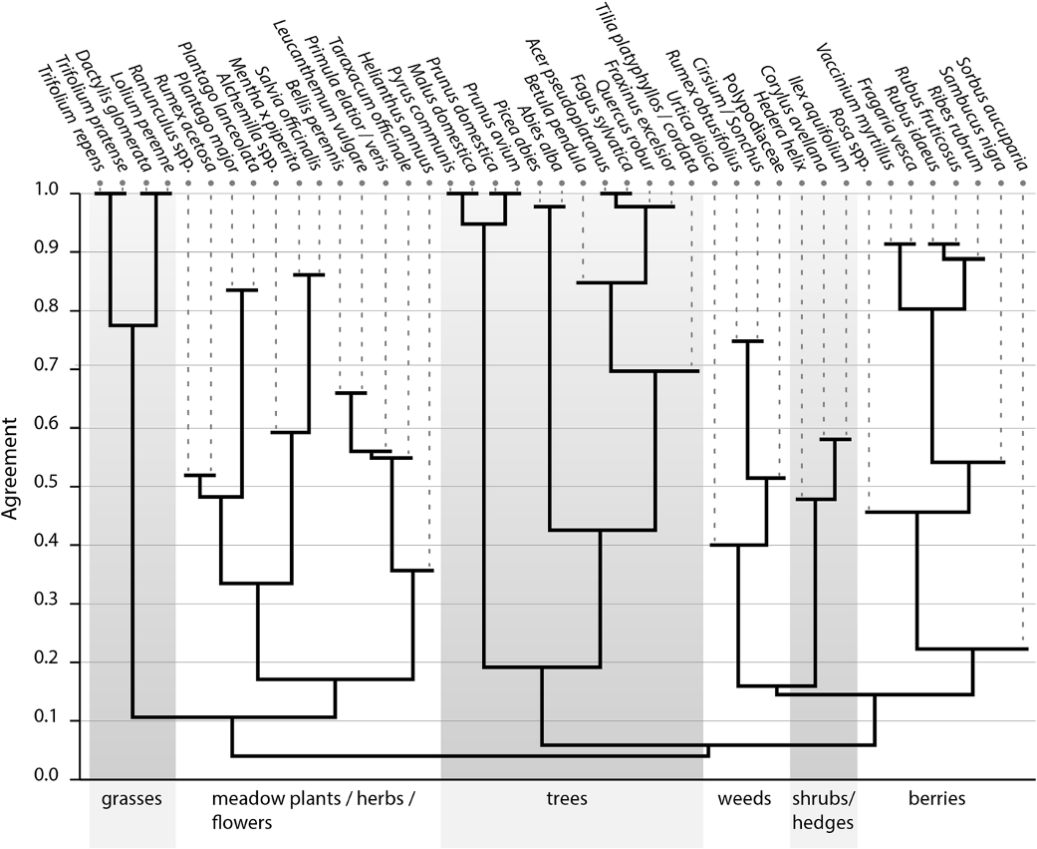
**Anthropac cluster analysis of the 36 pile sortings, groups indicated with generally given names (n = 36).**

Cultural Consensus Analysis did not detect significant variation among the informants (pseudo-reliability 0.990, first eigenvalue ratio 36.256) and the cluster diagram reveals six major groups (Figure 5). The groups are indicated with the name generally given by the informants: grasses, meadow plants/herbs/flowers, trees, weeds, shrubs/hedges, and berries.

Grasses are a well-defined, clearly separated group including the four most valuable forage grasses. It was sometimes split into clover (*Trifolium repens, T. pratense)* and grasses (*Dactylis glomerata, Lolium perenne*).

The meadow-group can be divided into three main subgroups. The first subgroup contains plants described as “not that good grasses” or “fill-ins”: *Ranunculus* spp., *Rumex acetosa, Plantago major, P. lanceolata*. They were by most informants perceived as neither good nor bad. The second subgroup contains the tea herbs *Alchemilla* spp*., Mentha piperita* and *Salvia officinalis*, the third subgroup the flowers *Bellis perennis, Leucanthemum vulgare, Primula elatior/veris, Taraxacum officinale* and *Helianthus annuus*. The three subgroups overlap one another and some plants were also put in other groups as the “weeds” or the “garden plants” (which do not appear as group in the cluster analysis). This explains the low coherence within the group.

Trees are a very well defined group, which is split into three subgroups. The first contains the fruit-trees pear, apple, prune and cherry (*Pyrus communis, Malus domestica, Prunus domestica, P. avium)*. The second contains the two conifers *Abies alba* and *Picea abies*, which supply also the best timber of the region. The third subgroups contains the deciduous trees of the forest (*Betula pendula, Acer pseudoplatanus, Fagus sylvatica, Quercus robur, Fraxinus excelsior, Tilia* spp*.*), where *Tilia* is weakly associated. The lime does not grow wild in the region, but is often planted next to the house or on top of hills. It was often put alone or together with the fruit trees.

Weeds are a small group consisting of *Urtica dioica, Rumex obtusifolius, Polypodiaceae,* and *Cirsium* spp. They are closely observed and eventually weeded. The stinging nettle *Urtica dioica* is rather weakly associated, because it was often put together with tea herbs.

The shrubs and hedges group contains *Hedera helix, Corylus avellana* and *Ilex aquifolium.* The ivy with its special growth form was difficult to group for the informants. It was often added only at the end of the task to the group which seemed most appropriate e.g. ornamental plants, hedges or the undergrowth of the forest. This group contained often also *Sambucus*, *Rosa* and *Sorbus*. But these three species were apparently more often defined by their fruits and appear therefore in the last group of the berries.

The berries group is defined by species with small, edible fruits, which are used to make jam and syrups: *Rosa* spp*., Vaccinium myrtillus, Fragaria vesca, Rubus idaeus, Rubus fruticosus, Ribes rubrum,* and *Sambucus nigra*. The rowan (*Sorbus aucuparia*) is weakly associated because although its fruits look similar to other berries, they are often supposed to be poisonous.

## Discussion

### Folkbotanical classification

The folkbotanical classification system of farmers’ families in the Napf region shows similar numbers of known plant taxa as those of indigenous societies all over the world [1]. The observed shallow taxonomic hierarchy with few higher order and many lower order taxa (generic, specific) is also typical for folkbotanical classification systems [1]. For the most important rank, the generic species, Berlin [1] extracted numbers from 17 ethnobotanical studies, ranging from 137 to 956 (80% monotypical), with traditional non-cultivators at the lower, and traditional cultivators at the upper end. With 316 generic taxa (85.4% monotypical), the present study lies in the lower range of the “traditional cultivators”. Typical for “traditional cultivators” is also the high amount of sub-generic taxa (e.g., different salads or forage grasses in our case), which is suspected to be driven by cultivation and related close observation of the plants.

The assignment of a taxon to a rank is generally difficult and prone to subjectivity. One of the encountered problems were primary names of specific or subspecific taxa. Primary, often simple names as “thistle” or “hazel” are usually a characteristic of generic taxa. There are, however, a few exceptions. A specific taxon may bear a primary name, if it is the prototype of the respective generic taxon [1], p.29. In our study, the generic taxon “*Hasle*” (*Corylus avellana*) is split into the two specific taxa “*Hasle*” and “*Zierhasle*”, whereby the former is the prototype and the latter a garden form of *Corylus avellana*. Abbreviation of complex specific or subspecific names may also occur [1], p.29. Our generic “Gras” (Poaceae) contains among many specifics of the type *“x-gras”* also the specific *“Timotee”* (*Phleum pratense*), which is an abbreviation of *“Timoteegras”.* We assigned also *“Fuchsschwanz”* (*Alopecurus* spp.), *“Trespe”* (*Bromus erectus*) and *“Goudhaber”* (*Trisetum flavescens*) to the generic *“Gras”,* although they lack the label *“-gras”* completely. But they were always listed or mentioned together with other grasses, never alone and – in the case of *“Goudhaber”* – never together with *“Haber”* (*Avena sativa*). It is also important to keep in mind, that plant names may have very different origins. Most of the grasses were listed and known only by men, especially by farmers, who had completed an agricultural school. From their training they knew the formal names of the grasses and pronounced them often in High German. Recently imported plant names like these cannot be expected to reflect a local classification system. The same is true for many garden plants, which often bear fanciful names.

The above mentioned *“Salat”* is another example of a folk generic containing several specifics which lack the epithet *“-salat”* and bear primary names. It could be argued that *“Gras”* and *“Salat”* are intermediate and, especially in the case of the salads, use-based taxa. We decided nevertheless to rank them as generics, because it needs expert knowledge to differentiate between the respective specifics. Less knowledgeable persons described the different specific taxa all as *“Gras”* or *“Salat”.* Furthermore, both names appeared frequently in freelists, where intermediate taxa are uncommon. Nevertheless, many of the generic, specific and subspecific taxa need further investigation to confirm the assigned rank.

The taxa of the intermediate level, which are after Berlin often covert, not named taxa [2], p. 26, were the most difficult to define and are certainly not complete. This is partly also due to the study design. While the open setting of the sorting task allowed identifying different important aspects of plant classification such as morphology, use and ecology (see below), a successive pile sorting [29], for example, would probably have forced the interviewees to classify in a more consequent and hierarchical manner.

### Plant knowledge

Several environmental education studies from industrialized countries show difficulties of young people to list plants. In Switzerland, over 6000 children and adolescents between the age of 8 and 16 listed on average five plants out of their immediate environment [11]. In Germany, only 14% of 3000 school leavers were able to list eight native wildflowers [36]. In South Carolina, USA, thirty-one 18-22-year-old college students freelisted in different local plant domains on average 9.0 crops, 8.4 trees, 5.4 garden flowers, 1.9 vines, 1.7 wildflowers/weeds and 1.4 grasses, so in total on average 27.8 plants [13]. Still in South Carolina, eleven 9-12-year-old children freelisted on average 30.9 plants in 10 different plant categories like trees, flowers, garden plants, shrubs etc. [37]. In Ajo, a rural town in Arizona, USA, 110 students between the ages of 12 and 20 freelisted on average five and in total 85 plants when asked to “name all of the plants that you know” [12].

In the Napf-region we found higher numbers of freelisted plants. Adults above 20 years listed on average 54 plants and even children and adolescents up to 20 years old listed on average 25 plants and in total 179 different plants. The average number given by children and adolescents is lower than in the two studies of South Carolina, but it was the outcome of only one freelist question. As freelists tend to be more complete the more focused the domain is [38], our number would probably be higher if we would have included more than one freelist question. The very unspecific life-form term “tree”, which was frequently mentioned in the above cited studies, did never appear in our freelists. “Grass” on the other hand was often mentioned, mostly by women and children, but meaning then only species of the families Poaceae, Cyperaceae and Juncaceae. It seems that even in industrialized countries, a rural population of small-scale farmers has enough direct contact with the surrounding environment to keep a respectable knowledge about plants.

While the “devolution” of plant knowledge seems less pronounced among rural people, it is still difficult to estimate how much formerly held knowledge has been eroded or changed. For example, the number of plants listed by the Napf children is modest compared to the 43 plant species known by 9 year old Tzeltal Maya children [39], although the difference may also be influenced by the plant diversity of the environment. In our data, we could not detect significant differences in the number of listed plants among older and younger adults. There was a significant increase of listed plants around the age of 20, which is probably due to professional specialization of the adults: Most men are educated farmers and most women manage a large homegarden.

A list of vernacular plant names gathered over the last 50 years in the north-eastern part of the Napf-region and the adjacent rural area of the canton of Lucerne (Amt Willisau) contains names of 653 species, split into 301 wild species, 81 crops and 301 fruit trees and garden plants [40]. Compared to our data (298 wild species, 213 cultivated plants), Brun-Hool reports much more names of cultivated plants, which might indicate a loss of knowledge in our area. However, while Brun-Hool is a specialist for homegardens, our freelist question was rather directed towards wild growing species, which may explain the above differences [41].

### Sorting criteria

Pile sorting is influenced by the (expertise) knowledge of the interviewee in different plant-related areas. Often different criteria are simultaneously used to explain plant (or fish) sorting [9, 14, 16, 42]. The more an individual knows about a plant, the less it uses only the most obvious morphological features for its assessment, or as Nolan puts it:

“… ethnobotanical classification is based fundamentally on the recognition of ostensible perceptual features of plants, but progressively guided by the recognition of culturally learned functional attributes.” [16] p. 69.

In our case this means that a basic morphological system is superposed by culturally influenced knowledge especially about use and habitat of the plants. Only our youngest informant, an eleven-years-old girl, sorted exclusively by morphology. Possibly she did not master additional knowledge to the same degree as others did. Interestingly, informants tended to switch criteria during one and the same pile sorting task. Apart from the unconstrained question the reason may lie in the fuzziness inherent to any given system. People used a new criterion as soon as a plant was difficult to classify.

The salience of morphology, use and habitat is underlined by other studies: all the three appear in an ethnomycological study from Indonesia and a folkbiological study of fish [43, 42], morphology and use in classification studies of trees and medicinal plants [14, 16], and morphology and habitat in an environmental education study [44]. A study from the French alps identified 23 “folk biotopes” and states that “folk botanical knowledge (…) is perceptually and practically linked to folk ecological knowledge, represented by the set of locally perceived higher order units analogous to the scientific ecologists’ habitat and biotop community” [17] p. 55. Additional criteria may emerge in other contexts. Seasonal aspects for example were important for sorting plants in a study in the Grosses Walsertal, Austria [45], personal communication as well as in the mentioned fish studies [9, 42].

Despite the open sorting task question, Cultural Consensus Analysis showed a high consensus among the informants and revealed no obvious subgroups or outliers. Even the four persons sorting consequently by a single criterion produced quite similar groups. Thus, the salient groups visible in the cluster analysis were the result of varying considerations. The tree group, for example, was explained mainly by morphology (“they are trees/they look alike”), but also by habitat (“they grow in the forest/in the tree garden”) or use (“we use the wood/the fruits”). The group of the grasses was explained mainly by use (“they are good fodder grasses”), but also by habitat (“they grow in meadows”) or morphology (“they are grasses/clovers). The two grasses as well as the two clover species were additionally strongly linked by their names. They were both called “–grass” (-*gras*: *Reigras*, *Chnoulgras*) and “–clover” (-*chlee*: *Wiiss*-*Chlee*, Rotchlee) respectively, which contributed probably to the fact, that they were never separated at all. The berries group was also quite stable, with slight variations: the description “berry” stressed the morphological aspect and included all plants with berry-like fruits, “berries in the garden” or “wild berries/berries in the forest” stressed the habitat and excluded *Sambucus nigra*, *Sorbus aucuparia*, *Vaccinium myrtillus*, *Fragaria vesca* and *Rosa* spp*.* in the first and *Ribes rubrum* in the second case, while “edible berries / you can make jam of them” stressed the use and excluded *Sorbus* and also *Rosa* for most of the people.

Obviously, in our study, “all roads lead to Rome” [14]. With another selection of pile sorting plants, this effect may have been weaker. But the plants forming the stable groups are good examples to demonstrate, that not only form and function [9, 14, 16], but also habitat and function [17] and, as known from plant ecology, habitat and form may be inherently linked. For example, trees are functional forms and the forest a functional habitat for people cutting and using wood – and forests are characterized by tree species. Grasses and clover are functional forms and meadows functional habitats for people breeding cattle – and meadows are characterized basically by grass species. For the berries, there was a particularly strong link between morphology and use. The term “berry” itself, which in botany is purely morphological, implies in the Napf region almost the edibility of the fruit. A young man explained his berry-group very typically like “Those are berries. Small, round and edible things”. He included *Sorbus aucuparia*, although he was in doubt about its edibility. But the local name “Vogubeeri”, literally “bird berry”, evocate that it is eaten at least by birds (and links it linguistically to the other berries). *Ilex aquifolium*, in contrast, appeared never in the berry group. Its local name “Stächpaume” contains no link to the berries and to edibility. The undeniably similar red fruits were only mentioned in an ornamental context.

### Different sorting criteria but similar groups

The phenomenon of similar sorting caused by different criteria has been frequently observed [9, 14, 16, 42]. Usually, the coincidence of form and function is explained by a certain form favouring a certain function [9] p. 875, [16] p. 77) or more generally by a “correlation of features that leads them [the informants] to form the same clusters” [14] p. 75. We here put this argument into perspective by using a more comprehensive perception of plant properties. This leads us to the field of sensory perception, which is known to be important for the classification of medicinal plants [46]. Plant perception, which leads to plant classification, happens not only visually, but through all our senses. Auditory perception plays certainly a minor role in the case of plants, but smell, taste and tactile perception are crucial to identify the qualities of a plant. As sight is the dominating human sense especially in western societies, reasoning about categories contains in many cases a strong morphological component. Evaluating plants by taste or smell results likely and directly in categories of use (or non-use). The berries, for example, were often put together because of their common use for jam or just because of their edibility. Behind this use-based explanation, a sensory perception argument is hidden: the berries are eaten or used to make jam, because they are all perceived as juicy, sweet and tasty. Other examples are *Mentha x piperita* and *Salvia officinalis*, which were rarely separated. They were said to be “kitchen herbs” or “tea herbs”, because they are perceived as fragrant species with physiological effects.

Since species of the same plant family tend to share as well morphological as chemical properties, perception with different senses may lead to similar groups. Using the visual and also tactile sense favours morphological explanations, while categories based on taste or smell are likely explained by use.

## Conclusions

Compared with urban populations of western societies, the rural population of the Napf region holds respectable plant knowledge. The folkbotanical classification system of the people is comparable to classification systems of indigenous societies, both in its shallow hierarchical structure and in the amount of recognized taxa.

The classification of plants was mainly guided by morphology, habitat and use. The three aspects may be mutually linked for certain plant groups, which results in always the same groups, independent from the different sorting criteria. Sensory perception allows for a broader explanation of the known coincidence of morphology and use groups. As related plant species share not only morphological, but also chemical properties, perception with different senses (visual, taste, smell) may lead to similar groups.

## Consent

Oral informed consent was obtained from the interviewees or their parents for the publication of this report.
